# Systemic administration of magnesium sulfate enhances lidocaine-induced sciatic nerve block and exerts analgesic effects in postsurgical pain in rats

**DOI:** 10.1186/s12871-026-03773-4

**Published:** 2026-03-21

**Authors:** Zhong-Mou Shi, Yun-Fei Lu, Yu Sun, Lu Chen, Fei Yang, Xiao-Zhi Wu

**Affiliations:** 1 Department of Anesthesiology, the First Affiliated Hospital, Fujian Medical University, Fuzhou, P. R. China; 2 Department of Anesthesiology, Binhai Campus of the First Affiliated Hospital, National Regional Medical Center, Fujian Medical University, Fuzhou, P.R. China; 3 Anesthesiology Research Institute, the First Affiliated Hospital, Fujian Medical University, Fuzhou, P. R. China; 4 Department of Anesthesiology and Perioperative Medicine, Fuzong Clinical Medical College of Fujian Medical University, 900th Hospital of PLA Joint Logistic Support Force, Fuzhou, P. R. China; 5 Department of Anesthesiology, Fifth Medical Center of PLA General Hospital, Beijing, P.R. China; 6 Pain Research Institute, Fujian Medical University, Fuzhou, P.R. China

**Keywords:** Magnesium sulfate, Postsurgical pain, Nerve block

## Abstract

**Purpose:**

Magnesium plays a role in various physiological processes and has been used for treatment and prevention of diseases affecting different organ systems. Magnesium sulfate has been proposed as an adjuvant for anesthesia, but its efficacy remains debated due to conflicting findings. The present study explored the analgesic effects of different routines of magnesium sulfate administration under three common perioperative situations in rats.

**Methods:**

The study tested several doses of magnesium sulfate (e.g., 30 mg/kg, 50 mg/kg) in rats under different perioperative conditions: (1) perineural co-administration of magnesium sulfate with lidocaine, (2) intraperitoneally magnesium sulfate pre-administration before lidocaine nerve block, and (3) systemic magnesium sulfate given before or after plantar incision surgery. Sensory/motor blockade duration and mechanical pain thresholds were assessed.

**Results:**

When magnesium sulfate is used as an adjunct to lidocaine, all doses of magnesium sulfate can reduce the sciatic nerve block caused by lidocaine. However, when administered via intraperitoneal injection, lower concentrations of magnesium sulfate (e.g., 30 mg/kg, 50 mg/kg) enhance the blocking effect of lidocaine, while higher concentrations (e.g., 150 mg/kg, 300 mg/kg) show no significant additional benefit. In a rat plantar incision model, both preoperative and postoperative intraperitoneal administration of magnesium sulfate effectively alleviated postoperative pain, with the 150 mg/kg dose yielding the most pronounced effect.

**Conclusion:**

Magnesium sulfate exhibits route-dependent effects: while it attenuates lidocaine-induced nerve blockade when administered locally, it enhances blockade duration and provides significant analgesia when given systemically. These findings implies that magnesium sulfate paves the way for designing safer nerve block protocols when administered locally, while its systemic application translates into more prolonged and superior postoperative analgesia, thereby reducing opioid consumption and facilitating patient recovery.

## Introduction

Magnesium is the fourth most abundant cation in the human body and the second most abundant intracellular cation after potassium. Magnesium plays a role in various physiological processes, including transmembrane ion flow, calcium channel gating, muscle contraction, cardiac excitability, vascular tone, neuronal activity, and neurotransmitter release [1, 2], whereas the disruption of magnesium homeostasis, typically hypomagnesemia, is related to various symptoms in the clinic [3]. The magnesium has been used for treatment and prevention of diseases affecting different organ systems. However, the efficacy of magnesium in most diseases has been controversial due to limited and conflicted previous findings.

Magnesium has also been reported to be widely used perioperatively. For example, in peripheral regional block techniques, magnesium sulfate has been used as an adjuvant to local anesthetics, whereas conflicting results have been reported. Some studies suggest that magnesium sulfate enhances nerve blockade, while others indicate it may attenuate local anesthetic effects under certain conditions. Clinical trials and in *vivo* animal studies have reported that when magnesium is used as an additive, it can prolong the duration of local anesthesia and provide better postoperative analgesia [4, 5]. However, other studies do not support this conclusion. A randomized controlled trial reported that adding magnesium sulfate did not improve the effect of adductor canal block after total knee arthroplasty [6]. A recent study by Hung et al. found that in *vivo*, magnesium sulfate shortened the duration of lidocaine-induced sciatic nerve blockade in rats, but whole-cell patch-clamp recordings in the same study showed the opposite result [7].

In addition to its role in peripheral nerve block, magnesium sulfate has also demonstrated its efficacy in managing postoperative pain. As a natural non-competitive N-methyl-D-aspartic acid (NMDA) receptor antagonist and calcium channel blocker, magnesium sulfate can inhibit calcium ion influx and reduce the excitability of NMDA receptors in the central nervous system, leading to decreased pain sensitivity in the central nervous system and achieving analgesic effects. This characteristic has prompted increasing research into the analgesic effects of magnesium sulfate in recent years. Previous studies have reported that magnesium sulfate can reduce acute postoperative pain and chronic neuropathic pain [8–10]. However, the optimal dosage and administration protocols to utilize these benefits remain unclear, and few clinical or preclinical studies have investigated whether aforehand administration of magnesium could alleviate or prevent postoperative pain.

Although magnesium has been widely used perioperatively, its efficacy remains controversial and the underlying mechanisms remains unknown due to limited investigations. In the present study, by mimicking the three common perioperative situations in the clinic, which conclude peripheral regional block, pre- and post- operation or the occurrence of the surgical incision, we investigated the effects of magnesium sulfate on lidocaine-induced sciatic nerve blockade through both local and systemic routines in a dose dependent manner, and the analgesic effects of magnesium sulfate on post-surgical pain at the timepoints of pre- or post-occurrence of the surgical incision. Hopefully, through the present study, we could provide more preclinical evidence of the application of magnesium in perioperative situations, like peripheral regional block and pain management.

## Materials and methods

### Animal

This study utilized 170 male Sprague-Dawley rats weighing 200–250 g, employing three distinct administration methods: (1) Perineural co-administration of magnesium sulfate and lidocaine; (2) Intraperitoneal injection of magnesium sulfate prior to lidocaine nerve block; (3) Intravenous injection of magnesium sulfate before and after plantar incision surgery. All rats were provided by the Laboratory Animal Center of 900th Hospital. Rats were housed five per cage under controlled temperature (23 ± 1℃) and humidity (50 ± 5%) with a 12 h light/12 h dark cycle and free access to food and water. All rats were acclimated in the test room for 5–7 d before behavioral experiments and all behavioral tests were conducted between 9:00 and 16:00 by using blind methods. Rats were randomly assigned throughout the whole trial. This study was conducted following the Animal Care and Use Committee of 900th Hospital (Authorization No.: 2021-006) and the National Institutes of Health guide for the care and use of laboratory animals (NIH Publications No. 8023, revised 1978). The number of rats used was minimized, as was their suffering.

### Rats sciatic nerve block model

Following the methods of previous sciatic nerve blockade models, rats were anesthetized with 2% isoflurane. The needle was inserted at a point one-third of the way along the line between the right greater trochanter and the ischial tuberosity, angled medially and anteriorly with the needle’s tail lifted approximately 45 degrees. Once the needle tip contacted bone, it was withdrawn by 1 mm, and then 0.5 ml of 0.5% lidocaine was administered. Immediately after the injection, the isoflurane inhalation was stopped, allowing the rats to recover from anesthesia, typically waking within about two minutes post-injection. Upon recovery, tactile, pinprick sensitivity, and motor functions were assessed immediately (starting at time zero). Assessments were then made at the fifth minute following recovery and subsequently every five minutes until full function was restored. Pinprick sensory block was evaluated by a 24-G needle as previously described and a successful pinprick block was defined as no response to the pinprick stimulus. Motor function was also assessed using the 0–3 scale. Motor function was quantified as 1 = normal motor function, 2 = normal dorsiflexion ability and the rat walking with curled toes, 3 = moderate dorsiflexion ability and the rat walking with curled toes, 4 = no dorsiflexion ability and the rat walking with curled toes. Sensory and motor function were evaluated every 30 min until the complete resolution of blockade.

### Mechanical pain sensitivity testing

Mechanical allodynia was measured using manual Von Frey filaments based on methods in published [11]. Rats were acclimatized for 30 min in plexiglass chambers (20 × 20 × 20 cm) with a metal mesh floor. Filaments (0.6–60 g bending force) were applied vertically to the hindpaw plantar surface from below the mesh. Stimulation began with the lowest force, progressively increasing. Each application lasted 5 s with > 15 s intervals between stimuli. Paw withdrawal, lifting, or licking constituted a positive response. Each filament was tested 10 times. The paw withdrawal mechanical threshold (PWMT, expressed in grams) was determined as the lowest filament force eliciting paw withdrawal responses in ≥ 50% of the applications (i.e., ≥ 5 responses out of 10 stimuli). The PWMT of bilateral hindpaws was measured.

### Incisional surgery

The postoperative pain model involving hind paw incisional surgery was developed as outlined in previous study [12]. Rats were anesthetized with 2% isoflurane in oxygen, delivered at a rate of 3 L/min during the procedure. The plantar aspect of the right hind paw was cleansed with 10% povidone-iodine solution before and after the operation and positioned through an aperture in a sterile surgical drape. A 1-cm incision was made longitudinally starting 0.5 cm from the heel’s proximal end towards the toes through the skin and fascia. The plantaris muscle beneath was then lifted and incised along its length, followed by suturing the skin with 5 − 0 nylon sutures. In contrast, sham-operated rats received anesthesia and their paws were treated with povidone-iodine without making an incision. Post-surgery, animals were placed in recovery cages and the incision was monitored daily for signs of infection or wound opening, with affected rats being removed from the study. Experiments commenced as soon as 2 h following the surgical procedure.

### Statistical analysis

Data are expressed as mean ± SEM. All data were statistically analyzed using SPSS 22.0 and GraphPad Prism 8.0. For normally distributed data, we used one-way/two-way ANOVA; for non-parametric data, Welch ANOVA was applied. The sample sizes were based on our previous knowledge and experience with this design. The normality test was performed by the Shapiro–Wilk test. The homogeneity of variance test was performed by Levene’s test. A level of *p*<0.05 were considered statistically significant.

## Result

### Magnesium sulfate attenuated lidocaine induced sciatic nerve blockade when applied as an adjuvant to lidocaine

**Fig. 1:**
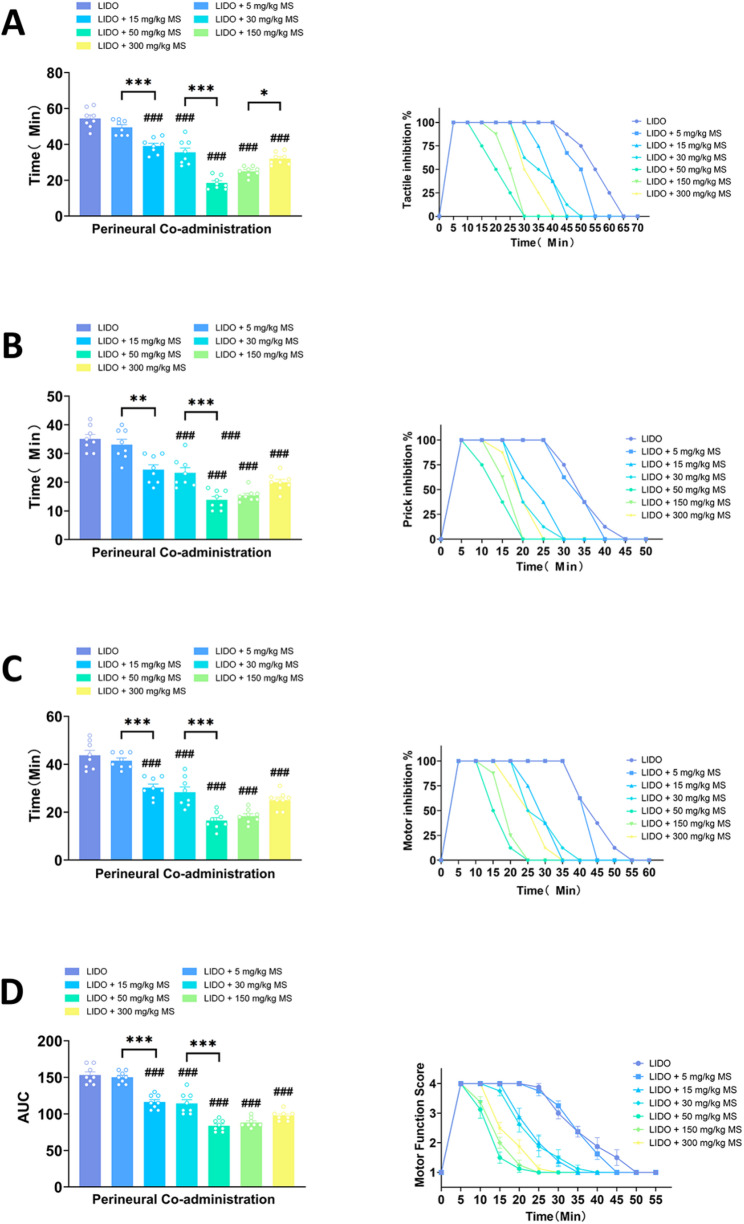
Magnesium sulfate attenuated lidocaine induced sciatic nerve blockade when applied as an adjuvant to lidocaine. **A** Durations of tactile blockade in different groups and the proportion of rats showing tactile inhibition over different time intervals represented as time curves. **B** Durations of pinprick nociceptive blockade in different groups and the proportion of rats showing pinprick inhibition over different time intervals represented as time curves. **C** Durations of motor blockade in different groups and the proportion of rats showing motor inhibition over different time intervals represented as time curves. **D** Time curves of motor blockade scores and the area under the curve (AUC) for each group. (n=8, ###*p* < 0.001 vs LIDO; *p < 0.05, **p < 0.01, ***p < 0.001 indicate differences between the groups. Data are expressed as mean ± sem).

As shown in Fig. 1, lidocaine induced sciatic nerve blockade in rats, exhibited as inhibited tactile, nociceptive and motor responses. When lidocaine was co-administrated with different concentrations of magnesium sulfate (5 mg/kg, 15 mg/kg, 30 mg/kg, 50 mg/kg, 150 mg/kg, 300 mg/kg), the durations of the inhibited tactile, nociceptive and motor responses were shortened significantly, compared to lidocaine alone induced sciatic nerve blockade (Table 1). It is notable that the attenuating effect of magnesium sulphate on lidocaine-induced nerve block did not follow a simple dose-dependent pattern. Within the low concentration range (5–50 mg/kg), the inhibitory effect gradually strengthened as the magnesium sulfate dose increased. However, within the high-concentration range (50–300 mg/kg), increasing the dose further resulted in a reduction of its inhibitory effect. At the lowest concentration tested (5 mg/kg), magnesium sulphate had no significant effect on lidocaine-induced nerve block. Overall, these findings suggest that magnesium sulfate’s modulatory effect on lidocaine-induced nerve block operates within an optimal dose range. The strongest inhibitory effect was observed at a dose of 50 mg/kg under the experimental conditions. Doses higher or lower than this concentration failed to achieve the same degree of attenuation, suggesting that the effect is only effective within a specific dose range and does not continually intensify with increasing dosage. This characteristic biphasic pattern suggests that the underlying mechanism may involve a complex interplay of multiple molecular targets (e.g., NMDA receptor antagonism and voltage-gated calcium channel blockade), with their net effect on neuronal excitability shifting as the local concentration of magnesium sulfate changes.

**Table 1:** One-Way ANOVA and pairwise comparisons

		P Value																				
	Function	Lido vs. 5	Lido vs. 15	Lido vs. 30	Lido vs. 50	Lido vs. 150	Lido vs. 300	5 vs. 15	5 vs. 30	5 vs. 50	5 vs. 150	5 vs. 300	15 vs. 30	15 vs. 50	15 vs. 150	15 vs. 300	30 vs. 50	30 vs. 150	30 vs. 300	50 vs. 150	50 vs. 300	150 vs. 300
**Perineural** **Co-administration**	Motor	0.9421	< 0.0001	< 0.0001	< 0.0001	< 0.0001	< 0.0001	< 0.0001	< 0.0001	< 0.0001	< 0.0001	< 0.0001	0.9759	< 0.0001	< 0.0001	0.2104	< 0.0001	0.0005	0.7083	0.9759	0.0047	0.0518
	Tactile	0.3089	< 0.0001	< 0.0001	< 0.0001	< 0.0001	< 0.0001	0.0006	< 0.0001	< 0.0001	< 0.0001	< 0.0001	0.6813	< 0.0001	< 0.0001	0.0413	< 0.0001	0.0005	0.7153	0.0811	< 0.0001	0.0475
	Prick	0.9998	< 0.0001	< 0.0001	< 0.0001	< 0.0001	< 0.0001	0.0022	0.0004	< 0.0001	< 0.0001	< 0.0001	> 0.9999	0.0001	0.0015	0.5748	0.0008	0.0084	0.9369	> 0.9999	0.0959	0.4753

### Systemic magnesium sulfate enhanced lidocaine induced sciatic nerve blockade

**Fig. 2:**
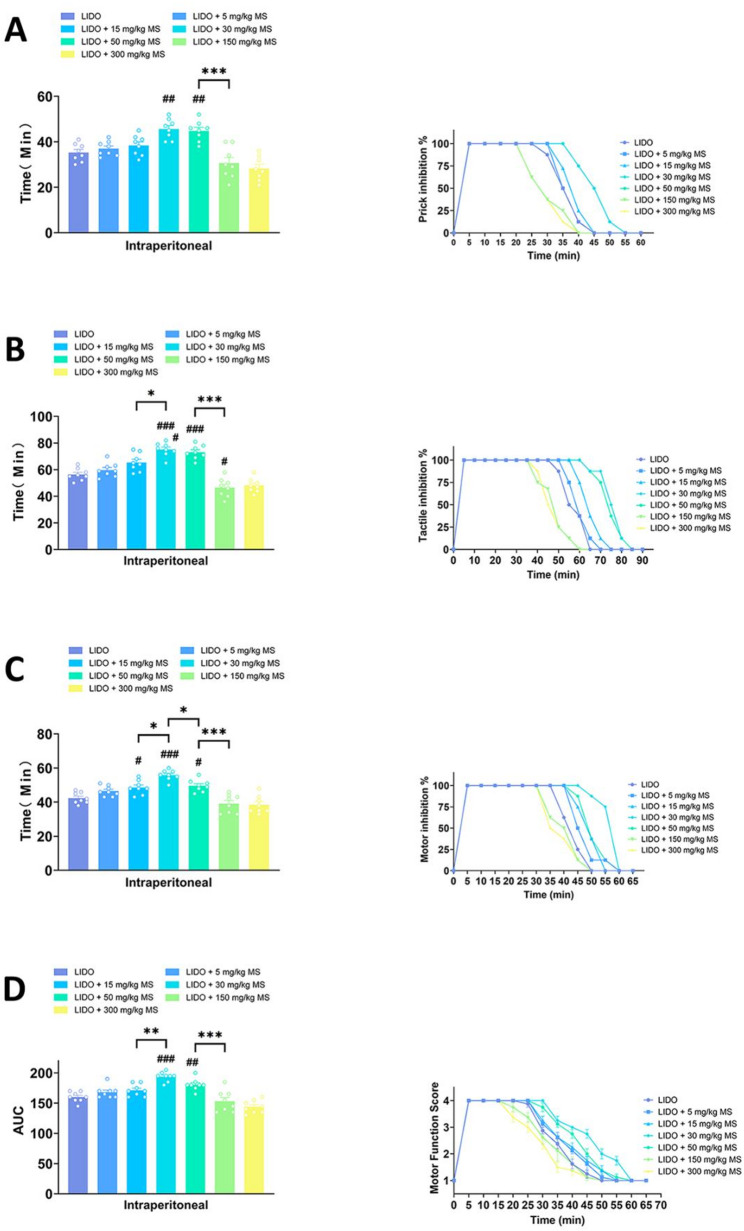
Systemic magnesium sulfate enhanced lidocaine induced sciatic nerve blockade. **A** Durations of tactile blockade in different groups and the proportion of rats showing tactile inhibition over different time intervals represented as time curves. **B** Durations of pinprick nociceptive blockade in different groups and the proportion of rats showing pinprick inhibition over different time intervals represented as time curves. **C** Durations of motor blockade in different groups and the proportion of rats showing motor inhibition over different time intervals represented as time curves. **D** Time curves of motor blockade scores and the area under the curve (AUC) for each group. (n=8, #*p* < 0.05, ##*p* < 0.01, ###*p* < 0.001 vs LIDO; *p < 0.05, **p < 0.01, ***p < 0.001 indicate differences between the groups. Data are expressed as mean ± sem)

In this part of the study, different concentrations of magnesium sulfate (5 mg/kg, 15 mg/kg, 30 mg/kg, 50 mg/kg, 150 mg/kg, 300 mg/kg) were injected intraperitoneally in rats prior to lidocaine induced sciatic nerve blockade. As shown in Fig. 2; Table 2, the enhancing effect of magnesium sulfate on lidocaine-induced blockade exhibited a clear dose-dependent relationship, yet with a distinct critical threshold. Specifically, only at doses of 30 mg/kg and 50 mg/kg did magnesium sulfate significantly prolong the duration of lidocaine-induced blockade in tactile, pain, and motor responses. It is noteworthy that doses below this range (e.g., 5 mg/kg and 15 mg/kg) showed no significant effect, while increasing the dose beyond this range to 150 mg/kg and 300 mg/kg did not result in any additional enhancement beyond that observed at 50 mg/kg. These findings indicate that the enhancing effect of magnesium sulfate operates within an optimal dose window, beyond which no further increase in efficacy is achieved.

**Table 2:** One-Way ANOVA and pairwise comparisons

		P Value																				
	Function	Lido vs. 5	Lido vs. 15	Lido vs. 30	Lido vs. 50	Lido vs. 150	Lido vs. 300	5 vs. 15	5 vs. 30	5 vs. 50	5 vs. 150	5 vs. 300	15 vs. 30	15 vs. 50	15 vs. 150	15 vs. 300	30 vs. 50	30 vs. 150	30 vs. 300	50 vs. 150	50 vs. 300	150 vs. 300
**Intraperitoneal**	Motor	0.39	0.0439	< 0.0001	0.0114	0.6668	0.4659	0.9358	0.0004	0.7057	0.0095	0.0038	0.0114	0.9987	0.0003	0.0001	0.0439	< 0.0001	< 0.0001	< 0.0001	< 0.0001	> 0.9999
	Tactile	0.8934	0.038	< 0.0001	< 0.0001	0.0166	0.0732	0.4346	< 0.0001	0.0004	0.0004	0.0027	0.0188	0.1098	< 0.0001	< 0.0001	0.9915	< 0.0001	< 0.0001	< 0.0001	< 0.0001	0.9973
	Prick	0.99	0.8475	0.0014	0.0043	0.4725	0.0731	0.9973	0.0124	0.0336	0.1315	0.0107	0.0569	0.1315	0.0336	0.0019	0.9998	< 0.0001	< 0.0001	< 0.0001	< 0.0001	0.9539

### Systemic magnesium sulfate alleviated postsurgical pain in a rat plantar incision model

**Fig. 3:**
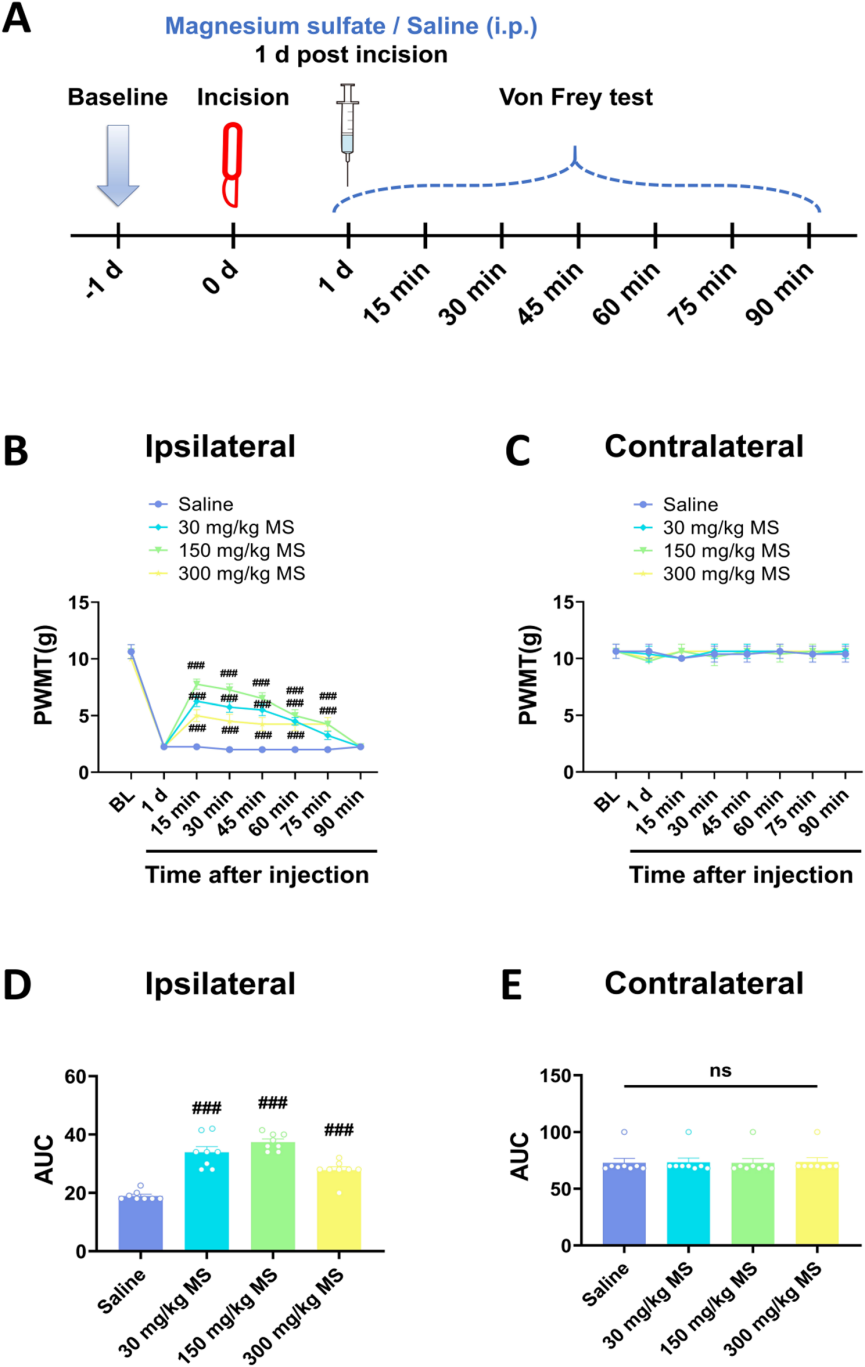
Post-operative intraperitoneal injection magnesium sulfate alleviated postsurgical pain in a rat plantar incision model. **A** Schematic diagram showing the procedure of the experiments evaluating the therapeutic effect of the single intraperitoneal injection of magnesium sulfate (30, 150, 300 mg/kg) on incision induced mechanical pain hypersensitivity. Magnesium sulfate is intraperitoneally injected 1 day after the incision surgery, and the von Frey test is performed at 15, 30, 45, 60, 75 and 90 min after the injection. **B**, **C** Time-course of the changes of the ipsilateral and contralateral paw withdraw mechanical threshold (PWMT) of different groups before and after magnesium sulfate injection.. n = 8, ###p < 0.001 vs Saline; two-way analysis of variance (ANOVA) of repeated measures followed by Dunnett’s post hoc test. **D**, **E** The area under curve (AUC) of the ipsilateral and contralateral PWMT. n = 8, ###p < 0.001 vs Saline, ns, no significance; one-way analysis of variance (ANOVA) of repeated measures followed by Tukey’s post hoc test. Data are expressed as mean ± sem.

**Fig. 4:**
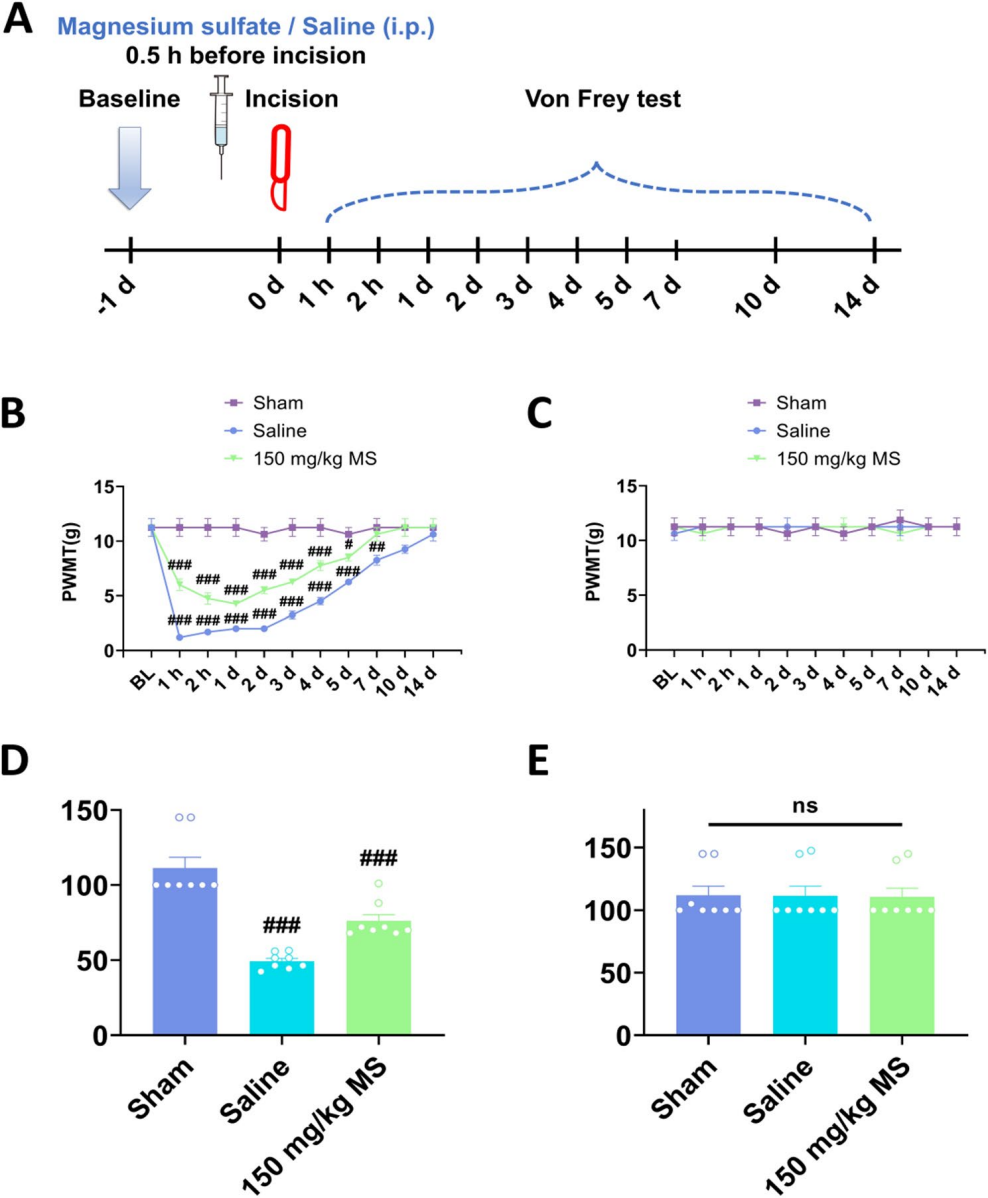
Pre-operative intraperitoneal injection magnesium sulfate attenuated and facilitated the recovery of postsurgical pain in a rat plantar incision model. **A** Schematic diagram showing the procedure of the experiments evaluating the preventive effect of the single intraperitoneal injection of magnesium sulfate (150 mg/kg) on incision induced mechanical pain hypersensitivity. Magnesium sulfate is intraperitoneally injected at 30 minutes before the incisional surgery, and the von Frey test is performed at 1, 2 h and 1, 2, 3, 4, 5, 7, 10, and 14 days after the incision. **B** and **C** The time-course of the changes of the ipsilateral and contralateral paw withdrawal mechanical threshold (PWMT). **D**, **E** The area under the curve (AUC) of the ipsilateral and contralateral PWMT. *n* = 8, ##*p* < 0.01, ###*p* < 0.001 vs Saline, ns, no significance; one-way analysis of variance (ANOVA) of repeated measures followed by Tukey’s post hoc test. Data are expressed as mean ± sem

To investigate the analgesic effects of magnesium sulfate, the rats received a plantar incision aforehand. One day post-surgery, the rats exhibited mechanical pain hypersensitivity in the hindpaw that received surgery and the mechanical pain threshold in the intact contralateral hindpaw did not show significant alterations. Following i.p. injection of different concentrations of magnesium sulfate (30 mg/kg, 150 mg/kg, and 300 mg/kg), the mechanical pain hypersensitivity was alleviated in the hindpaw that received surgery, whereas the mechanical pain threshold in the intact hindpaw remained relatively unchanged. The analgesic effects of magnesium lasted more than 75 min, and did not show any dose dependency, with the most prominent analgesic effects observed at the dosage of 150 mg/kg (Fig. 3). Furthermore, to investigate whether magnesium sulfate had preventive effects on postsurgical pain, the rats received i.p. injection of magnesium sulfate before they were subjected to the plantar incision surgery. Following the plantar incision surgery, the rats showed mechanical pain hypersensitivity, however, the decrement of mechanical pain threshold in the magnesium sulfate treated rats were relatively smaller than saline control (Fig. 4). Moreover, the mechanical pain hypersensitivity in the rats that received i.p. magnesium sulfate injection lasted 7 days, which lasted 14 days in the saline control rats (Fig. 4). The magnesium sulfate not only alleviated but also facilitated the recovery of the postsurgical pain.

## Discussion

Magnesium ion is an important electrolyte that involved in major cellular and physiological processes. Although many advances have been made in the understanding the roles of magnesium sulfate in clinical medicine, information regarding the regulatory processes of this cation at cellular, tissue and systems levels is still lack. Moreover, some evidence concerning the efficacy of magnesium sulfate are controversial and thus warrants further study. In the present study, we investigated the effects of magnesium sulfate on lidocaine-induced sciatic nerve blockade and post-surgical pain, by mimicking the three common perioperative situations in the clinic, which conclude peripheral regional block, pre- and post- operation or the occurrence of the surgical incision. We found that, magnesium sulfate attenuated lidocaine induced sciatic nerve blockade when applied as an adjuvant to lidocaine, whereas systemic administration of magnesium sulfate enhanced lidocaine induced sciatic nerve blockade. Systemic administration of magnesium sulfate, either before or after the plantar incision was made, alleviated the degree and duration of the postsurgical pain in a rat plantar incision model.

Consistent with the findings of Hung et al., our study also observed that co-administration of magnesium sulfate with lidocaine shortened the duration of sciatic nerve blockade in rats. However, while their study reported a dose-dependent inhibitory effect of magnesium sulfate, our results demonstrated a biphasic dose-response relationship, with the most pronounced inhibition occurring at 50 mg/kg [7]. These differing outcomes are thought to be due to the concentration of lidocaine used in this study, which was 0.5%, compared to the 2% employed by Hung et al. This significant difference in the baseline anaesthetic concentration may have resulted in nerve membrane potentials and sodium channel availability being in different states. Under the subsaturated blockade condition with 0.5% lidocaine, magnesium sulphate may more readily modulate nerve conduction through its calcium channel-blocking and NMDA receptor-antagonising effects. Conversely, the modulatory scope may be limited in the near-saturated blockade induced by 2% lidocaine, thereby exhibiting a different dose-response pattern.

The precise mechanism by which magnesium sulfate shortens the duration of peripheral nerve blockade remains incompletely understood. Local anesthetics primarily act by blocking voltage-gated sodium channels (VGSC) on neuronal axons. This blockade reduces the probability of VGSC activation and prolongs the refractory period by slowing the recovery from the inactivated to the resting state [13]. We hypothesize that the interaction between magnesium sulfate and lidocaine involves several potential mechanisms related to VGSC function. First, the differential sensitivity of VGSC subtypes to magnesium may be a key factor. In primary sensory neurons, VGSC subtypes exhibit distinct properties and expression patterns [14, 15]. For instance, the NaV1.6 subtype, which contributes to tetrodotoxin-sensitive currents [16, 17], has been shown by Vastani et al. to be partially blocked by magnesium sulfate [18]. Furthermore, studies indicate that magnesium sulfate exerts more significant effects on Aβ fibers than on C-fibers. This selectivity could be attributed to the fact that tetrodotoxin-resistant currents in C-fibers are predominantly mediated by NaV1.8 and NaV1.9 subtypes [19], which may be less susceptible to magnesium. As peripheral nociception primarily relies on Aδ and C-fibers [20], the differential blockade of VGSC subtypes could partly explain the modulatory, rather than profound analgesic, effect of magnesium sulfate and its ability to shorten the non-selective blockade by lidocaine. Second, a biophysical mechanism may involve the modulation of the local electric field. According to the surface charge theory [21], divalent cations like Mg^2+^can theoretically shield negative surface charges on the neuronal membrane, potentially influencing the gating of VGSC. This interaction could alter the conformation and availability of VGSC, thereby modifying the binding and/or efficacy of lidocaine. Finally, a potential pharmaceutical interaction must be considered. The pH of magnesium sulfate solution is approximately 6, lower than that of lidocaine (pH 7.8). This difference could, in theory, influence the degree of ionization of lidocaine, potentially reducing the concentration of the active base form. However, as noted by Hung et al., the rapid buffering capacity of tissue fluid (pH 7.4) at the injection site might minimize this effect in vivo [7]. Whether this factor contributes to the observed shortening of the blockade warrants further investigation. In summary, the shortening of lidocaine-induced nerve blockade by magnesium sulfate is likely a complex phenomenon arising from its potential to differentially modulate VGSC subtypes and alter the local membrane environment, the net effect of which is influenced by the baseline anesthetic state established by lidocaine.

ing with the results mentioned above, that magnesium sulfate shortened the duration of lidocaine induced sciatic nerve block in rats, when magnesium sulfate was co-administered with lidocaine through perineural injection [7], clinical trials in humans, as well as in *vivo* animal investigations showed that magnesium sulfate can prolong the duration of regional anesthesia and provide better postoperative analgesia, when used as an additive, and applied through intravenous or intrathecal routine [22–24]. The discrepancies of the efficacy of magnesium sulfate may be attributed to the differences in the action sites, peripheral vs. systemic or central nervous system. The present study also found that systemic administration of magnesium sulfate enhanced lidocaine induced sciatic nerve blockade, and exerted analgesic effects on postsurgical pain in a plantar incision model in rats, suggesting its enhancing or analgesic effects might primarily come from systemic action. One mechanism of its analgesic action may involve NMDA receptor antagonism, which prevents central sensitization triggered by peripheral noxious stimuli and reduces acute pain following tissue injury. This mechanism is also relevant for local anesthetic adjuvants [25]. Additionally, magnesium sulfate’s analgesic effects may involve calcium channels in the dorsal root ganglia. Magnesium sulfate acts as a physiological calcium antagonist [26], regulating neuronal excitability, plasticity, and pathological changes [27]. Kido et al. used a skin/muscle incision and retraction (SMIR) model of chronic postoperative pain [28], found that preoperative injection of magnesium sulfate into loose skin on the neck, which routine also leads to systemic absorption, reduced SMIR-induced mechanical allodynia, possibly by regulating glutamate ionotropic receptor NMDA type subunit 1a (*Grin1*) expression. Similarly, in our study, pre-incisional magnesium sulfate administration alleviated postoperative pain and facilitated its recovery. Additionally, magnesium ions, as a second messenger, play a relatively novel signaling role compared to calcium ions (Ca²⁺) and other second messengers [29]. Intracellular magnesium levels critically regulate key signaling pathways, such as ERK, CREB, and mTOR [30]. The ERK/CREB pathway, central to pain pathogenesis, mediates pain through oxidative stress, neuroinflammation, neurodegeneration, and glial activation [31]. The mTOR pathway, activated after nerve injury, regulates downstream molecules like NPY in sensory neurons, contributing to neuropathic pain development [32]. These findings provide hints for further research investigating the mechanisms underlying the analgesic effects of magnesium sulfate.

There are several limitations in the present study that should be acknowledged. First, the rat plantar incision model employed in this study primarily mimics acute postoperative pain caused by tissue injury. Therefore, the analgesic efficacy and mechanisms observed here may not be directly extrapolated to other clinical scenarios, such as chronic postoperative pain or neuropathic pain, which involve distinct pathophysiological processes. Second, while we discussed potential mechanisms involving voltage-gated sodium channels (VGSCs) and signaling pathways (e.g., ERK/CREB), our conclusions are based on behavioral phenotypes and existing literature. We did not provide direct experimental evidence, such as patch-clamp electrophysiological recordings of VGSC activity or Western blot analysis of protein expression/phosphorylation levels (e.g., NaV1.6, p-ERK, p-CREB). Third, regarding the interaction between lidocaine and magnesium sulfate, we hypothesized mechanisms related to channel blockade. However, we did not experimentally evaluate the specific effect of this drug combination on the steady-state inactivation of sodium channels. Future studies utilizing electrophysiological and molecular techniques are warranted to further elucidate these underlying mechanisms. Finally, while the present study focused exclusively on the pharmacological profile of magnesium sulfate, we did not compare its efficacy with other commonly used adjuvants, such as epinephrine, dexmedetomidine, or clonidine. Future studies comparing magnesium sulfate with these established agents are warranted to better position its role in clinical practice.

In conclusion, by mimicking the three common perioperative situations, the present study found that the effects of magnesium sulfate on lidocaine-induced sciatic nerve blockade in rats varied by administration routine: local application attenuated lidocaine induced sciatic nerve blockade, while systemic application enhanced it. Systemic magnesium sulfate, either before or after the occurrence of postsurgical pain, alleviated the degree and duration of the postsurgical pain in a rat plantar incision model. These findings provide critical guidance for the clinical use of magnesium sulfate: when employed as an adjunct to regional anesthesia, the distinct outcomes of local versus systemic administration must be strictly differentiated; while simultaneously affirming systemic magnesium sulfate as a promising multimodal analgesic strategy for the perioperative period. Its value lies in the potential to reduce opioid consumption and achieve sustained analgesic benefits that encompass both preoperative prevention and postoperative treatment.

## Data Availability

Data and materials are available from the corresponding authors upon reasonable request.

## References

[1] Dubé L, Granry JC. The therapeutic use of magnesium in anesthesiology, intensive care and emergency medicine: a review. Can J Anaesth. 2003;50(7):732–46. 12944451 10.1007/BF03018719

[2] Gao Q, Cil O. Magnesium for disease treatment and prevention: emerging mechanisms and opportunities. Trends Pharmacol Sci. 2024;45(8):708–22. 39019764 10.1016/j.tips.2024.06.004 PMC11892326

[3] Touyz RM, de Baaij JHF, Hoenderop JGJ. Magnesium Disorders. N Engl J Med. 2024;390(21):1998–2009. 38838313 10.1056/NEJMra1510603

[4] Zeng J, Chen Q, Yu C, Zhou J, Yang B. The Use of Magnesium Sulfate and Peripheral Nerve Blocks: An Updated Meta-analysis and Systematic Review. Clin J Pain. 2021;37(8):629–37. 34128482 10.1097/AJP.0000000000000944

[5] Kroin JS, McCarthy RJ, Von Roenn N, Schwab B, Tuman KJ, Ivankovich AD. Magnesium sulfate potentiates morphine antinociception at the spinal level. Anesth Analg. 2000;90(4):913–7. 10735798 10.1097/00000539-200004000-00025

[6] Zoratto D, Phelan R, Hopman WM, Wood GCA, Shyam V, DuMerton D, Shelley J, McQuaide S, Kanee L, Ho AM, et al. Adductor canal block with or without added magnesium sulfate following total knee arthroplasty: a multi-arm randomized controlled trial. Can J Anaesth. 2021;68(7):1028–37. 34041719 10.1007/s12630-021-01985-5

[7] Hung YC, Chen CY, Lirk P, Wang CF, Cheng JK, Chen CC, Wang GK, Gerner P. Magnesium sulfate diminishes the effects of amide local anesthetics in rat sciatic-nerve block. Reg Anesth Pain Med. 2007;32(4):288–95. 17720112 10.1016/j.rapm.2007.03.008 PMC2001297

[8] Srebro D, Vuckovic S, Milovanovic A, Kosutic J, Vujovic KS, Prostran M. Magnesium in Pain Research: State of the Art. Curr Med Chem. 2017;24(4):424–34. 27978803 10.2174/0929867323666161213101744

[9] Kulik K, Żyżyńska-Granica B, Kowalczyk A, Kurowski P, Gajewska M, Bujalska-Zadrożny M. Magnesium and Morphine in the Treatment of Chronic Neuropathic Pain-A Biomedical Mechanism of Action. Int J Mol Sci. 2021;22(24):13599. 10.3390/ijms222413599 PMC8707930 34948397

[10] De Oliveira GS Jr., Castro-Alves LJ, Khan JH, McCarthy RJ. Perioperative systemic magnesium to minimize postoperative pain: a meta-analysis of randomized controlled trials. Anesthesiology. 2013;119(1):178–90. 23669270 10.1097/ALN.0b013e318297630d

[11] Deuis JR, Dvorakova LS, Vetter I. Methods Used to Evaluate Pain Behaviors in Rodents. Front Mol Neurosci. 2017;10:284. 28932184 10.3389/fnmol.2017.00284 PMC5592204

[12] Guo ZB, Tang L, Wang LP, Wu HH, Huang CL, Zhan MX, Shi ZM, Yang CL, Chen GZ, Zou YQ, et al. The analgesic effects of ulinastatin either as a single agent or in combination with sufentanil: A novel therapeutic potential for postoperative pain. Eur J Pharmacol. 2021;907:174267. 34146590 10.1016/j.ejphar.2021.174267

[13] Catterall WA. Forty Years of Sodium Channels: Structure, Function, Pharmacology, and Epilepsy. Neurochem Res. 2017;42(9):2495–504. 28589518 10.1007/s11064-017-2314-9 PMC5693772

[14] Tan ZY, Wu B, Su X, Zhou Y, Ji YH. Differential expression of slow and fast-repriming tetrodotoxin-sensitive sodium currents in dorsal root ganglion neurons. Front Mol Neurosci. 2023;16:1336664. 38273939 10.3389/fnmol.2023.1336664 PMC10808659

[15] Hoffmann T, Kistner K, Nassar M, Reeh PW, Weidner C. Use dependence of peripheral nociceptive conduction in the absence of tetrodotoxin-resistant sodium channel subtypes. J Physiol. 2016;594(19):5529–41. 27105013 10.1113/JP272082 PMC5043034

[16] Katz E, Stoler O, Scheller A, Khrapunsky Y, Goebbels S, Kirchhoff F, Gutnick MJ, Wolf F, Fleidervish IA. Role of sodium channel subtype in action potential generation by neocortical pyramidal neurons. Proc Natl Acad Sci U S A. 2018;115(30):E7184–92. 29991598 10.1073/pnas.1720493115 PMC6065046

[17] Caldwell JH, Schaller KL, Lasher RS, Peles E, Levinson SR. Sodium channel Na(v)1.6 is localized at nodes of ranvier, dendrites, and synapses. Proc Natl Acad Sci U S A. 2000;97(10):5616–20. 10779552 10.1073/pnas.090034797 PMC25877

[18] Vastani N, Seifert B, Spahn DR, Maurer K. Sensitivities of rat primary sensory afferent nerves to magnesium: implications for differential nerve blocks. Eur J Anaesthesiol. 2013;30(1):21–8. 23138572 10.1097/EJA.0b013e32835949ab

[19] Clare JJ. Targeting voltage-gated sodium channels for pain therapy. Expert Opin Investig Drugs. 2010;19(1):45–62. 20001554 10.1517/13543780903435340

[20] Colloca L, Ludman T, Bouhassira D, Baron R, Dickenson AH, Yarnitsky D, Freeman R, Truini A, Attal N, Finnerup NB, et al. Neuropathic pain. Nat Rev Dis Primers. 2017;3:17002. 28205574 10.1038/nrdp.2017.2 PMC5371025

[21] Catterall WA. Cellular and molecular biology of voltage-gated sodium channels. Physiol Rev. 1992;72(4 Suppl):S15–48. 1332090 10.1152/physrev.1992.72.suppl_4.S15

[22] Telci L, Esen F, Akcora D, Erden T, Canbolat AT, Akpir K. Evaluation of effects of magnesium sulphate in reducing intraoperative anaesthetic requirements. Br J Anaesth. 2002;89(4):594–8. 12393361 10.1093/bja/aef238

[23] Brill S, Sedgwick PM, Hamann W, Di Vadi PP. Efficacy of intravenous magnesium in neuropathic pain. Br J Anaesth. 2002;89(5):711–4. 12393768

[24] Bilir A, Gulec S, Erkan A, Ozcelik A. Epidural magnesium reduces postoperative analgesic requirement. Br J Anaesth. 2007;98(4):519–23. 17324976 10.1093/bja/aem029

[25] Verma V, Rana S, Chaudhary SK, Singh J, Verma RK, Sood S. A dose-finding randomised controlled trial of magnesium sulphate as an adjuvant in ultrasound-guided supraclavicular brachial plexus block. Indian J Anaesth. 2017;61(3):250–5. 28405040 10.4103/ija.IJA_466_16 PMC5372407

[26] Fawcett WJ, Haxby EJ, Male DA. Magnesium: physiology and pharmacology. Br J Anaesth. 1999;83(2):302–20. 10618948 10.1093/bja/83.2.302

[27] Hartung JE, Moy JK, Loeza-Alcocer E, Nagarajan V, Jostock R, Christoph T, Schroeder W, Gold MS. Voltage-gated calcium currents in human dorsal root ganglion neurons. Pain. 2022;163(6):e774–85. 34510139 10.1097/j.pain.0000000000002465 PMC8882208

[28] Kido K, Katagiri N, Kawana H, Sugino S, Konno D, Suzuki J, Yamauchi M, Sanuki T. Effects of magnesium sulfate administration in attenuating chronic postsurgical pain in rats. Biochem Biophys Res Commun. 2021;534:395–400. 33246558 10.1016/j.bbrc.2020.11.069

[29] Stangherlin A, O’Neill JS. Signal Transduction: Magnesium Manifests as a Second Messenger. Curr Biol. 2018;28(24):R1403–5. 30562536 10.1016/j.cub.2018.11.003

[30] Yamanaka R, Shindo Y, Hotta K, Suzuki K, Oka K. GABA-Induced Intracellular Mg(2+) Mobilization Integrates and Coordinates Cellular Information Processing for the Maturation of Neural Networks. Curr Biol. 2018;28(24):3984–e39913985. 30528584 10.1016/j.cub.2018.10.044

[31] Zhen W, Zhen H, Wang Y, Chen L, Niu X, Zhang B, Yang Z, Peng D. Mechanism of ERK/CREB pathway in pain and analgesia. Front Mol Neurosci. 2023;16:1156674. 37008781 10.3389/fnmol.2023.1156674 PMC10060514

[32] Chen L, Hu Y, Wang S, Cao K, Mai W, Sha W, Ma H, Zeng LH, Xu ZZ, Gao YJ et al. mTOR-neuropeptide Y signaling sensitizes nociceptors to drive neuropathic pain. JCI Insight. 2022;7(22):e159247. 10.1172/jci.insight.159247 PMC9746821 36194480

